# On Chinese learning strategies of learners from Central Asian countries: an analysis of gender, age, and learning duration effects

**DOI:** 10.3389/fpsyg.2024.1372005

**Published:** 2024-05-09

**Authors:** Ruiling Chen, Lirui Zhang

**Affiliations:** School of Foreign Languages, Xuchang University, Xuchang, Henan, China

**Keywords:** Chinese learning strategies, Central Asian learners, gender, age, learning duration

## Abstract

This research focuses on the Chinese learning strategies employed by learners from Central Asian countries, specifically examining the effects of gender, age, and learning duration. The study aims to identify and analyze the demographic factors that influence the learning approaches of these learners, providing insights for more effective teaching and learning of Chinese as a foreign language. Data are collected through questionnaires and interviews, and statistical analysis is conducted to explore the correlations between gender, age, learning duration, and learning strategies. The learning strategy model adopted in this study provides a comprehensive classification of language learning strategies. The results underscore the significance of incorporating these factors into language education programs, providing valuable insights into the unique needs and challenges encountered by learners from Central Asian countries. The findings indicate that students from Central Asian countries predominantly utilize social strategies, metacognitive strategies, and affective strategies in their Chinese language learning. These are followed by compensatory strategies, cognitive strategies, and memory strategies. While gender does not exert a significant impact on the utilization of Chinese learning strategies, there are discernible variations in memory and affective strategies between males and females. Age does not significantly affect overall learning strategies, but there are notable disparities in compensatory strategies among different age groups. Learning duration has a significant effect on compensation and metacognitive strategies. The correlation between learning duration and overall strategies is significant indicating that learners with different learning durations exhibit notable differences in compensation and metacognitive strategies.

## Introduction

Among the various tools that enhance second language (L2) learning, learning strategies are considered to be the most important and essential techniques that learners can use to improve their L2 achievements (Al-Khazale, 2019). Research has shown that the use of learning strategies can facilitate skill acquisition, boost confidence, and foster motivation among language learners (Nguyen and Godwyll, 2010; Chai et al., 2016). The efficacy of language learning strategies is subject to individual variability, including factors such as age, gender, and duration of study. Accurately discerning the efficacious learning strategies and delineating which strategies should be employed by learners with distinct individual characteristics to augment L2 acquisition outcomes constitutes a significant challenge within the domain of L2 acquisition research. Consequently, there has been a growing interest in understanding the factors that influence learners’ learning strategies and outcomes (Khamkhien, 2010; Radwan, 2011; Alhaysony, 2017; Corbin, 2017; Zhang and Wan, 2019). However, most studies have focused on English education due to its status as a *lingua franca* in second language education (Radwan, 2011; Chou, 2018; Gong et al., 2020; Zhang et al., 2022; Ibrahim et al., 2023). In contrast, there is a lack of research on L2 Chinese acquisition despite the increasing number of international students studying Chinese as a foreign language worldwide (Ding, 2016; Wang, 2017; Li et al., 2021; Zhang and Tsung, 2021; Min et al., 2022). With the ongoing implementation of the “Belt and Road” initiative, China has witnessed closer exchanges and collaboration with Central Asian countries. This has also created more opportunities for learners from Central Asian (hereinafter referred to as the subjects) to pursue their education in China. Hence, it is crucial to examine the Chinese learning strategies adopted by these students who come to China for education.

Identifying the factors that influence learning strategies is essential for effective teaching and learning of Chinese as a foreign language (CFL). By examining how gender, age, and learning duration^1^ impact students’ learning strategies, educators can develop more effective teaching methods and tailor their approaches to meet the diverse needs of their students (Khamkhien, 2010; Radwan, 2011; Alhaysony, 2017; Corbin, 2017; Zhang and Wan, 2019). Additionally, this research may contribute to a deeper understanding of the complexities involved in CFL acquisition and provide insights into the ways in which learners from different cultural backgrounds approach language learning.

## Literature review

### Learning strategies

Learning strategies have become increasingly important in the learning process, as they play a crucial role in enhancing its effectiveness, self-direction, and enjoyment. This importance is underscored by various scholars who have contributed to our understanding of these strategies. Oxford (1989) was foundational in defining learning strategies as the actions or behaviors utilized by learners to improve their language learning experience. Building on this definition, Pressley and McCormick (1995) added that these strategies are techniques for comprehending, retaining, and applying information that are intentionally employed and consciously managed by the learner.

Furthermore, Macaro (2006) emphasized that learning strategies are cognitive in nature and cannot be solely considered in terms of observable behavior, suggesting a deeper mental dimension to these strategies. This cognitive perspective is echoed by Zhang et al. (2022), who describe language learning strategies as conscious mental and behavioral procedures that individuals engage in to gain control over their learning process. Ortega (2009) defined them as deliberate goal-directed attempts to manage and control efforts to learn the second language (L2), highlighting the proactive and intentional aspect of these strategies.

In a similar vein, Brown (2006) described them as procedures that facilitate a learning task, while Griffiths (2008) stated that they are activities consciously chosen by learners to regulate their language learning. Taken together, these scholarly views paint a comprehensive picture of learning strategies as multifaceted, encompassing both cognitive and behavioral elements, and emphasizing the learner’s intentional and conscious engagement in the learning process.

### Language learning strategies

Within the realm of learning strategy research, the language learning strategies (LLS) encompass the deliberate techniques deployed by learners to facilitate the acquisition, retention, storage, usage, and retrieval of a new language. Oxford (1990) views these strategies as instrumental in fostering communicative competence. Acknowledging the critical influence of learning strategies on foreign language acquisition, a multitude of studies have been conducted to unravel their intricacies (Rafik-Galea et al., 2011; Oxford, 2013; Szyszka, 2017). Among these, the specific strategies that students adopt when developing second language (L2) skills have garnered particular attention (Oxford, 2013; Szyszka, 2017). Milla and Gutierrez-Mangado (2019) meticulously documented the LLS preferences of bilingual learners adept in Basque and Spanish. Their findings indicated that irrespective of proficiency levels or gender, minimal variance was observed in the range or adoption of LLS among the participants. Chen’s (2014) exhaustive investigation examined the LLS employed by English as a Foreign Language (EFL) learners across diverse educational tiers, revealing significant correlations between various age groups and their proclivity for employing memory, compensation, metacognitive, and affective strategies in language learning. Empirical evidence underscores the importance of adept learning strategy use for language learners to advance their proficiency. Nevertheless, further exploration is warranted to discern which learning strategies are most efficacious and suitable for addressing specific language tasks.

In order to better illustrate language learning strategies, the following topic will focus on the classification of language learning strategies.

### Classification of language learning strategies

Since the 1980s, scholars have systematically categorized language learning strategies, with Rubin (1975), Bialystok (1978), and O’Malley and Chamot (1990) offering distinct classifications informed by cognitive theory. These early classifications laid the foundation for understanding how learners process, retain, and use language information, providing crucial insights into the cognitive mechanisms underpinning language acquisition.

Building upon the seminal work of Oxford (1990), the classification of language learning strategies has garnered widespread acceptance and has been extensively incorporated into the research endeavors of numerous scholars (Aslan, 2009; My and Dan, 2023). In this vein, Psaltou-Joycey et al. (2014) delved into the LLS preferences of a cohort of 103 Greek primary school students pursuing English as a foreign language. Their study was anchored on an adapted version of Oxford’s (1990) Strategy Inventory for Language Learning (SILL) questionnaire, customized to resonate with the Greek educational context. The findings disclosed that the average scores for four out of the six strategic categories were within a moderate range, whilst metacognitive and affective strategies were consistently favored. In a related study, Alhaysony (2017) investigated the LLS employed by Saudi EFL learners at Aljouf University, utilizing a variant of Oxford’s (1990) SILL questionnaire. The results indicated that the frequency of strategy use among the participants was generally in the low to medium bracket, with cognitive, metacognitive, and compensation strategies being the most frequently utilized, contrasting with memory and affective strategies which were the least commonly employed.

### Previous studies on the effects of gender, age, and learning duration on L2 learning strategies

The impact of gender on language learning strategies has been examined in the field of Second Language Acquisition (Oxford and Nyikos, 1989; Griffiths, 2003; Aslan, 2009; Nguyen and Godwyll, 2010; Sumarni and Rachmawaty, 2019; Melvina Lengkanawati and Wirza, 2020; Montero-SaizAja, 2021; Dinsa et al., 2022). Oxford and Nyikos (1989) reported that females utilized cognitive, metacognitive, and social strategies more frequently than males in their study of undergraduate learners of foreign languages. However, Nyikos (1990) noted that males made greater use of specific strategies in an investigation into possible test type bias in tests of recall among university level beginner learners of German. Ehrman and Oxford (1989) found that female learners used strategies for searching, functional practice strategies, self-management strategies, and communicating meaning more than males. Gender disparities are also evident in the strategies employed by males and females. Female learners tend to utilize more conversational and input strategies (Oxford and Nyikos, 1989), social learning strategies (Ehrman and Oxford, 1989), and memory and metacognitive strategies (Khalil, 2005) than their male counterparts. Montero-SaizAja (2021) revealed that females used language learning strategies significantly more than males, but there were no statistically significant differences between them regarding productive vocabulary. Similarly, Dinsa et al. (2022) denoted that there were significant relationships between females and males in employing speaking strategies to learn speaking skill. Melvina Lengkanawati and Wirza (2020) also found no significant differences between the two groups of students based on their gender, while Chang (2003) found that gender differences were significant in the use of cognitive, compensation, metacognitive, and social strategies, but were not detected in the use of memory and affective strategies.

Research has explored how age affects the use of learning strategies among language learners. According to Chen’s (2014) study, there are statistically significant relationships between different age groups and their preference for various learning strategies. Older learners tend to favor strategies that emphasize social interaction and practical functionality. Conversely, Milla and Gutierrez-Mangado’s (2019) research found that younger students, such as those in grade 5, reported using remembering strategies more frequently than those in grade 6. Additionally, Nguyen and Godwyll (2010) observed differences in strategy use across different age groups, indicating that age plays a role in the adoption of learning strategies.

The length of time a student has been studying a language also influences their choice of learning strategies. Oxford and Nyikos (1989) found that students who had studied a language for more than 5 years used functional practice strategies more often than less experienced students. Similarly, Dinsa et al. (2022) reported a significant difference between second and third-year EFL students in their use of speaking strategies to improve speaking proficiency. Chou (2018) discovered that full-time students used compensation and social speaking strategies more than part-time students to enhance their oral skills. Yaman and Ozcan (2015) noted a disparity in the application of speaking strategies between senior and junior students, indicating that study duration affects strategy use. However, Chang’s (2003) investigation found no significant differences in the use of learning strategies among students of different ages or with varying years of English study. This suggests that while there is generally a positive correlation between the length of study and strategy use, there may be exceptions to this trend.

From previous research, we can conclude that some studies identified differences related to gender, age, and learning duration in language learning strategies, while others did not. These findings suggest that factors such as gender, age, and learning duration may influence the choice of strategies used by learners. However, further investigation is required to fully understand these relationships. The current investigation explores the impact of demographic factors on the learning strategies employed by learners from Central Asian nations, aiming to offer valuable insights that can enhance the efficacy of teaching and learning Chinese as a foreign language. The study’s specific objectives are:

1. To analyze the Chinese language learning strategies adopted by the subjects.
2. To identify and examine the demographic variables, namely gender, age, and duration of study, that shape the learning preferences of these individuals.
3. To offer recommendations and provide actionable insights aimed at optimizing Chinese language education for the subjects.

## Conceptual framework

This study is rooted from Oxford’s (1990) classification, which divides language learning strategies into two main classes, direct and indirect, which are further subdivided into six groups. Direct strategies in language learning are those that learners employ directly with the target language, facilitating the acquisition and use of the language itself. Indirect strategies play an important role in language learning as they complement direct strategies by providing support and guidance to learners, helping them to develop effective learning habits and strategies. These strategies include:

1.   Memory Strategies: techniques used to link new information with existing knowledge, aiding memorization. This may include methods such as: creating mental linkages, applying images and sounds, reviewing well, and Employing action.
2.   Cognitive Strategies: mental operations that help learners understand and retain language material. Some examples are: practicing, receiving and sending messages, analyzing and reasoning, and creating structure for input and output.
3.   Compensation Strategies: approaches used to overcome limitations in knowledge or performance. They include: guessing intelligently, and overcoming limitations in speaking and writing.

These direct strategies are essential for learners as they engage directly with the language, helping them to acquire, comprehend, and use the target language effectively. Indirect strategies in language learning are those that help learners to understand and use the target language more effectively, but do not involve direct interaction with the language itself. These strategies include:

4.   Metacognitive Strategies: these are strategies that involve planning, monitoring, and evaluating one’s own learning process. Examples of metacognitive strategies include: creating a study plan to organize learning activities, self-testing to assess understanding and progress, and reflecting on the learning process to identify areas for improvement.
5.   Affective Strategies: these strategies aim to manage emotions, motivation, and attitudes towards language learning. Some examples of affective strategies include: reducing anxiety by managing expectations and focusing on positive outcomes, encouraging self-confidence through self-praise and positive self-talk, and maintaining a positive attitude towards learning by focusing on the benefits and enjoyment of language learning.
6.   Social Strategies: these strategies involve communication and collaboration with others to facilitate language learning. Examples of social strategies include: asking questions to clarify meaning and seek clarification from others, collaborating with peers to practice speaking and listening skills, and imitating the pronunciation and intonation of native speakers to improve fluency.

Oxford’s framework not only aids in the understanding of language learning strategies but also serves as a practical tool for educational planning and research design, contributing significantly to the field of language education.

## Methodology

To achieve these objectives, this study employs a mixed-methods approach, combining both quantitative and qualitative data collection methods. Data is collected through self-report questionnaires and face-to-face interviews. The questionnaires assess participants’ demographic information, including gender, age, and learning duration, as well as their use of various Chinese language learning strategies. The interviews provide further insights into participants’ experiences and perspectives on their language learning processes.

### Participants

All participants of this study are CFL learners from five Central Asian countries who are currently studying at five universities in mainland China. The valid participants are 110 learners from Kazakhstan (*N* = 57), Uzbekistan (*N* = 13), Kyrgyzstan (*N* = 9), Turkmenistan (*N* = 16), and Tajikistan (*N* = 15). At the time of the study the participants’ ages were 18 to 39 years old (mean of 24.75, *SD* = 6.75). Of these participants, 67 were males (60.9%), and 43 were females (39.1%). On average, they had been learning Chinese for 2.07 years and their proficiency level could be roughly described as upper intermediate, with some degree of individual variation.

From this pool of participants, we meticulously selected 30 individuals who are representative in terms of gender, age, and duration of study. This subset was chosen for in-depth interviews, aiming to ensure that our findings are reflective of the broader population of CFL learners from Central Asia within the context of mainland China. The criteria for selection were as follows:

1. Demographic representation: Interviewees were chosen to proportionally mirror the gender, age, and country of origin of the entire participant sample. This included individuals from each of the five Central Asian countries, maintaining a similar male-to-female ratio as observed in the main study (approximately 60–70% males to 30–40% females), and spanning the age range of 18 to 39 years old.
2. Proficiency and duration of study: The selected interviewees exhibit a variety of proficiency levels within the upper intermediate category, taking into account individual variability, and include participants who have been studying Chinese for varying lengths of time within the average range (approximately 2.07 years). This approach allows us to explore the impact of these factors on language acquisition and cultural adaptation.
3. Voluntary participation and unbiased sampling: All interviewees volunteered for the study and provided informed consent. Where feasible, random sampling methods were employed to minimize selection bias, ensuring that the sample accurately represents the broader population of CFL learners from Central Asia studying in mainland China. All participants were thoroughly briefed on the research objectives, methodologies, and potential implications. They were also asked to provide written informed consent, emphasizing their right to withdraw from the study at any point without any adverse consequences. Pseudonyms have been assigned to protect the identities of the interviewees.

### Instrumentation

The present study employed a questionnaire based on the Language Learning Strategy Inventory (SILL) developed by Oxford (1990), which classifies language learning strategies into two primary categories and six subcategories: direct strategies (cognitive strategies, compensation strategies, memory strategies) and indirect strategies (metacognitive strategies, social strategies, affective strategies). The questionnaire consisted of 30 questions, with each of the six strategies evenly distributed across the questions and each sub-item covering five questions. The questionnaire consists of two parts. The first part collects demographic information about the respondents, such as their gender, age, and years of Chinese learning. The second part involves adapting the content of SILL to fit the characteristics of Chinese learning and then utilizing the five-point Likert scale to finalize the questionnaire design. Respondents are asked to rate their agreement with each statement on a scale from 1 (Never or almost never true of me) to 5 (Always or almost always true of me). To ensure the reliability and validity of the questionnaire, we carried out a small-scale pilot test with 15 students and refined the questionnaire content based on the participants’ responses. The content of the questionnaire is shown in Appendix I.

This study utilizes Thematic Analysis as a tool for qualitative data examination. This technique is employed to recognize, assess, and report patterns (themes) within the collected data. It serves to mitigate the researcher’s preconceptions, permitting the inherent patterns of the data to steer the analytical process. Consequently, this method facilitates the extraction of salient themes that offer insight into the participants’ experiences, emotions, and strategies utilized in learning Chinese, thereby providing an extensive understanding of the participants’ perspectives. The interviews conducted are face-to-face, semi-structured, and chiefly designed to evoke profound insights into the participants’ experiences and viewpoints concerning their language acquisition journey. The interview content is documented in Appendix II.

### Procedure

The survey is conducted online in the form of “Questionnaire Star.” After designing the questionnaire online, it is sent to the participants through WeChat or QQ links. A total of 115 questionnaires are retrieved, and the retrieval rate is 95.6%. After the exclusion of 5 invalid questionnaires, 110 remain for data analysis. Zhang and Wan (2019) have conducted a thorough analysis of the questionnaire survey data. Subsequently, targeted interviews are administered grounded in the preliminary findings. Informed by the synergistic results of both the questionnaire and interview data, the study has formulated pedagogical recommendations tailored for the cohort under investigation. To be noted, in order to gain a comprehensive understanding of the interviewee’s viewpoint, we have chosen five Chinese learners with high proficiency from the five Central Asian countries. These individuals will be responsible for conducting interviews in their native languages with participants from their respective countries and subsequently translating the interview content into Chinese.

In order to quantify and evaluate the frequency and effectiveness of various language learning strategies employed by the subjects, statistical analysis is conducted. The study utilizes descriptive statistics, such as mean and standard deviation, as well as inferential statistics, including correlation analysis and regression analysis, to identify the most frequently utilized strategies. This approach aims to address Objective 1 by determining the optimal language learning strategies for the population under study.

Quantitative data analysis is employed to investigate the influence of demographic variables, such as gender, age, and duration of study, on language learning strategy preferences. Inferential statistical methods, including *t*-tests or analysis of variance (ANOVA), are utilized to discern differences in strategy utilization across distinct genders or age cohorts. Furthermore, correlational analysis is applied to assess the relationship between the duration of study and the propensity for certain learning strategies. This methodological approach is designed to fulfill Objective 2 by identifying the demographic factors that significantly impact the selection of language learning strategies.

Based on the outcomes of the quantitative data analysis, targeted recommendations are posited. For instance, if the data indicates a significant correlation between a particular learning strategy and enhanced language proficiency, the study may advocate for the integration of this method into teaching practices. Additionally, by delineating the learning preferences associated with specific demographic groups, the research can inform the development of tailored educational curricula, thereby accommodating the distinct needs of these cohorts. This approach is anticipated to effectively address Objective 3.

In the qualitative data analysis phase of this study, we commence with an immersive familiarization process, whereby all interview transcripts are read and recordings listened to multiple times, allowing for a nuanced understanding of the participants’ experiences. This is followed by an open coding step, in which relevant segments of the data are marked with codes derived from the context itself, ensuring a direct linkage to the participants’ actual narratives. Patterns and connections between these codes are then sought, leading to the formation of themes by grouping related codes together, a task that involves careful comparison and contrasting to discern their relationships. The potential themes that emerge are reviewed and refined to ensure internal consistency and distinctiveness, with each theme closely tied back to the research questions. Finally, each theme is clearly named to reflect its underlying essence, accompanied by detailed analyses that articulate the theme’s connection to the study’s research questions. This methodical approach to qualitative analysis complements the quantitative data, providing a comprehensive exploration of the participants’ perspectives on learning Chinese, thus offering a multifaceted view of the broader language learning experience.

## Results and discussion

Before starting the investigation, the reliability and validity of the scale are initially examined. Cronbach’s alpha coefficient is employed as the internal consistency index of the scale to assess its reliability, and a correlation analysis is conducted on the bivariate variables. The findings of the reliability coefficient and the correlation coefficient between each subscale and the overall scale, along with the significance analysis, are presented in Table 1.

**Table 1:** Reliability assessment.

Scale		Reliability coefficient	Bivariate analysis
Overall scale		0.926	——
Subscales	Cognitive	0.691	0.845 (*p* < 0.01, very significant)
	Compensatory	0.690	0.743 (*p* < 0.01, very significant)
	Memory	0.720	0.788 (*p* < 0.01, very significant)
	Metacognitive	0.842	0.849 (*p* < 0.01, very significant)
	Social	0.716	0.831 (*p* < 0.01, very significant)
	Affective	0.737	0.797 (*p* < 0.01, very significant)

As shown in Table 1 above, this scale is suitable for measuring the learning strategies of CFL learners, and it has good internal consistency. The results of bivariate correlation analysis show that this scale also has high homogeneity.

This research utilizes factor analysis to assess the structural validity of six scales, and the results are detailed in Table 2.

**Table 2:** Validity assessment.

	Strategies	Communality (%)	KMO	*p*-value (Significance)
Direct strategies	Cognitive	45.19	0.738	0.000
	Compensatory	45.82	0.719	0.000
	Memory	48.16	0.737	0.000
Indirect Strategies	Metacognitive	61.45	0.819	0.000
	Social	47.68	0.696	0.000
	Affective	49.57	0.758	0.000

Factor analysis results indicate that the communalities of the six scales are all above 0.45. Additionally, KMO statistics are mostly above 0.7, and *p*-values are all below 0.001, signifying high structural validity for the scales.

According to scale development standard proposed by Shi et al. (2012), 10 experts were invited to assess the content validity from tow perspectives of item-level content validity index (I-CVI) and scale-level content validity index based on the average (S-CVI/Ave). The results showed that I-CVI > 0.8 and S-CVI/Ave = 0.95, the content validity is satisfying.

### General condition

Generally, the statistical data reveals that the three indirect learning strategies with higher average scores among participants are social strategies (*M* = 3.91), metacognitive strategies (*M* = 3.80), and affective strategies (*M* = 3.75). Conversely, the three direct strategies exhibit slightly lower scores, ranking from highest to lowest as compensation strategies (*M* = 3.60), cognitive strategies (*M* = 3.47), and memory strategies (*M* = 3.38). The results of the analysis of variance on the mean scores of the subjects across each subscale indicate a highly significant main effect of the strategy [*F*(5,654) = 7.117, *p* < 0.01].

In addition, the results of pairwise multiple comparisons reveal that participants’ average scores in the social, metacognitive, and affective strategies are significantly higher than their scores in the cognitive and memory strategies (*p* < 0.05). However, no significant difference exists among the three direct strategies (*p* > 0.05). The compensatory strategies also demonstrate a significant advantage over the memory strategies (*p* > 0.05). Nevertheless, the disparities between the compensatory and the cognitive strategies, as well as between the cognitive and memory strategies, are not statistically significant (*p* > 0.05). The statistical findings are presented in Table 3.

**Table 3:** General condition.

	Strategies	*N*	Mean	Standard deviation	Maximum	Minimum
Direct strategies	Cognitive	110	3.4691	0.8001	5.00	1.60
	Compensatory	110	3.6018	0.8039	5.00	1.00
	Memory	110	3.3745	0.8704	5.00	1.00
Indirect strategies	Metacognitive	110	3.8018	0.8574	5.00	1.40
	Social	110	3.9145	0.7367	5.00	1.00
	Affective	110	3.7491	0.8011	5.00	1.00

The data presented in Table 3 reveals that the most frequently employed learning strategies by the subjects are social strategy, metacognitive strategy, and affective strategies, followed by compensatory, cognitive, and memory strategies. Notably, the first three strategies are classified as indirect strategies, while the latter three are categorized as direct strategies. This aligns with previous research on language learners’ strategy use (Vidal, 2002; Chen, 2014; Al-Khazale, 2019; My and Dan, 2023), which highlights the importance of social and metacognitive strategies. However, our study also emphasizes the role of affective strategies and raises questions about the effectiveness of memory strategies for this particular group of learners. A further comparison with previous studies reveals some discrepancies. The present study found that affective strategies were used frequently, while Al-Khazale’s (2019) study did not mention it. The primary reason for this difference in conclusions is the variation in the nationality of the subjects. Our study focuses on learners from five Central Asian countries, while Al-Khaza’le’s paper examines Saudi EFL learners in Saudi Arabia. This suggests that national factors should be considered in language acquisition research. In terms of least frequently used strategies, the present study found that memory strategy was used with low frequency, while compensation strategies score the lowest mean among the six learning strategies according to My and Dan (2023). It is possible that the students in our study had difficulty remembering new vocabulary or grammar rules, leading to a lower usage of memory strategy.

The observed patterns in strategy selection can be elucidated by examining the unique attributes and educational contexts of the subjects. Qualitative data derived from participant interviews indicate that these individuals often exhibit extroverted behaviors, characterized by openness, enthusiasm, and advanced communicative capabilities. They frequently leverage their acquired Chinese language proficiency through interactions with native speakers. Moreover, the study’s participants are adult learners, with the youngest subject being at least 18 years of age, possessing robust cognitive skills, mature thought processes, and well-developed self-monitoring and self-regulation capacities. Consequently, these adult learners demonstrate the ability to modulate their learning attitudes and emotional responses during the language acquisition process, proactively mitigating any anxieties associated with learning Chinese. Furthermore, their heightened interest in China, the Chinese language, and its culture serves as a catalyst for eager engagement with the Chinese populace. Thus, it is posited that social strategies, metacognitive strategies, and affective strategies represent the triad of most frequently employed learning strategies among the subjects under scrutiny.

### Correlation between genders and learning strategies

The mean and standard deviation of the subjects of different genders on each subscale are shown in Table 4.

**Table 4:** Mean and standard deviation of different genders on each subscale.

	Strategies	Gender	*N*	Mean	Standard Deviation	Maximum	Minimum
Direct strategies	Cognitive	Male	67	3.3970	0.63	1.60	5.00
		Female	43	3.5814	0.68	2.00	5.00
	Compensatory	Male	67	3.5910	0.83	1.00	5.00
		Female	43	3.6186	0.47	2.00	5.00
	Memory	Male	67	3.2418	0.52	1.00	5.00
		Female	43	3.5814	0.64	2.20	5.00
Indirect strategies	Metacognitive	Male	67	3.8179	0.51	1.40	5.00
		Female	43	3.7767	0.55	1.60	5.00
	Social	Male	67	3.8507	0.80	1.00	5.00
		Female	43	4.0140	0.90	2.20	5.00
	Affective	Male	67	3.6209	1.08	0.00	5.00
		Female	43	3.9488	0.91	2.40	5.00
Overall strategies		Male	67	3.5866	0.43	2.19	5.00
		Female	43	3.7535	0.38	2.47	4.80

In terms of mean scores, females exhibit superior performance compared to males in all subscales except for metacognitive strategies. The overall mean score for females is 3.75, which surpasses the male score of 3.59. To delve deeper into the gender disparities, a variance analysis was conducted on each subscale, as illustrated in Table 5.

**Table 5:** Variance analysis of different genders on each subscale.

	Strategies	*F*-value	*P*-value (Significance)
Direct strategies	Cognitive	1.394	0.240
	Compensatory	0.031	0.862
	Memory	4.101	0.045
Indirect strategies	Metacognitive	0.060	0.087
	Social	1.289	0.259
	Affective	4.531	0.036
Overall strategies		1.704	0.195

The results of the analysis of variance indicate that the main effect of gender on overall strategies is not statistically significant [*F*(1, 108) = 1.704, *p* > 0.05]. This suggests that there is no substantial difference in the overall strategies employed by individuals of different genders. However, when scrutinizing the subscales, the findings reveal that gender has a significant impact on memory strategies and affective strategies (*p* < 0.05), implying that notable disparities exist in these strategies between males and females. Additionally, the two-variable correlation analysis reveals that the correlation between gender and overall strategies is not statistically significant (*p* > 0.05). Nevertheless, the correlation between gender and memory strategies as well as affective strategies is statistically significant (*p* < 0.05), with correlation coefficients of 0.191 and 0.20, respectively.

Overall, the present study reveals no significant gender disparities in the utilization of Chinese learning strategies among the subjects. The selection of learning strategies by male and female learners is predominantly similar. Nevertheless, a close examination of the subscales indicates that there are pronounced discrepancies in memory strategies and affective strategies between male and female students. Notably, the positive correlation coefficient signifies that females tend to employ these two strategies more frequently than males. This finding concurs with Melvina Lengkanawati and Wirza’s (2020) research, which also reported no significant differences between male and female students in their use of cognitive, compensation, metacognitive, and social strategies, but did uncover gender variations in the application of memory and affective strategies. This outcome aligns with previous studies, which suggest that female learners often employ a greater number of strategies compared to their male counterparts (Ehrman and Oxford, 1989; Oxford and Nyikos, 1989; Khalil, 2005). Due to physiological differences, males tend to have inferior language memory and imitation abilities compared to females. Furthermore, the present study reveals that females are more inclined to employ affective strategies in their language learning, which is consistent with Bacon (1992) assertion that females tend to possess lower self-confidence but exhibit more positive affective reactions than males. As a result, females generally exhibit greater sensitivity and emotional reactivity compared to males, making them more adept at utilizing self-encouragement, mental adjustment, and other affective methods when acquiring languages.

To further explore the selection of memory strategies and affective strategies between males and females, we conducted interviews, and two exemplary cases are presented below. It is noteworthy that pseudonyms have been assigned to the interviewees to ensure the protection of their identities. Bo, a 39-year-old male learner from Kyrgyzstan, explicitly stated during the interview that he favors the use of cognitive strategies and social strategies over memory strategies. Bo, a 39-year-old male learner from Kyrgyzstan, was selected for his divergent perspective on strategy use, which aligns with our quantitative findings that males tend to favor cognitive and social strategies over rote memorization. He explicitly stated during the interview:

1.   Compared to simply memorizing facts and details, I prefer to gain true understanding and ability through thinking, exploration, and practice. For me, learning is an active process. I enjoy exploring new knowledge and perspectives through reading, discussing, and problem-solving. I believe that through communication and interaction with others, I can better understand and apply the knowledge I have learned. Instead of rote memorization, I prefer to combine knowledge with real-life situations to cultivate my critical thinking and problem-solving skills. Of course, I also understand that memory strategies are necessary in certain situations. Sometimes, when faced with a large amount of information or complex knowledge points, simple memorization may be more efficient and practical. However, I do not believe that relying solely on memory strategies is the only way to learn. I believe that everyone has their own unique learning style and methods, and it is important to find a way that suits you to enhance your learning effectiveness.

Another male learner, Ho from Kazakhstan, was chosen to represent those who reported minimal influence from affective strategies. His narrative supports our qualitative observations that males often rely on intrinsic motivation and self-drive rather than external emotional support:

2.   In spite of my anxiety, I will not let it affect me. I have faith in my abilities and potential, and I am confident that with hard work and determination, I can overcome obstacles and make progress. While encouragement and support from others are motivating, I will not rely on them excessively. Instead, I prioritize internal motivation and self-drive, believing that I can conquer challenges. When it comes to language learning, emotional adjustment is not my primary concern; instead, I will focus on learning methods and techniques to find a suitable approach to learning.

Ho clearly states that although he feels anxious, he will not let this emotion affect him and emphasizes that while encouragement and support from others can indeed motivate him, he does not overly rely on these external factors. Instead, he prioritizes intrinsic motivation and self-drive, believing in his ability to conquer challenges. Regarding language learning, He believes that emotional adjustment is not his main concern. He concentrates on learning methods and techniques, seeking a learning approach that suits him. Ho’s attitude and behavior indicate that he is self-sufficient in terms of emotional strategies, and not greatly influenced by external factors. He relies on his own confidence, intrinsic motivation, and focuses on cognitive and technical learning strategies, rather than depending on emotional support or emotional management.

The two viewpoints mentioned above are highly representative and provide a clear explanation for why males tend to choose memory strategies and affective strategies less frequently than females. This also offers valuable insights for targeted teaching in the classroom.

### Correlation between age groups and learning strategies

Age is identified as a prominent factor influencing the employment of learning strategies across numerous scholarly works (Bialystok, 1997; Nguyen and Godwyll, 2010; Chen, 2014; Milla and Gutierrez-Mangado, 2019; Dinsa et al., 2022). In terms of age categorization, Bialystok (1997) conducted research on the efficacy of foreign language learning across various age groups and concluded that “the age threshold with substantial disparities in language proficiency is 20 years old.” Consequently, we have divided the participants into four groups: 18–20 years old, 21–30 years old, 31–38 years old, and above 39 years old, based on the age boundaries of 20 and 30. The mean values and standard deviations for each subscale among international students of different ages are presented in Table 6.

**Table 6:** Mean and standard deviation of different age groups on each subscale.

	Strategies	Age groups	*N*	Mean	Standard deviation	Maximum	Minimum
Direct strategies	Cognitive	18–20	54	3.5000	0.7758	1.60	5.00
		21–30 31–38	46 7	3.5217 2.8571	0.8159 0.4995	1.80 2.20	5.00 3.60
		Over 39	3	3.5333	1.3614	1.60	4.60
	Compensatory	18–20	54	3.7148	0.8062	1.00	5.00
		21–30 31–38	46 7	3.6174 2.9714	0.7931 0.5701	1.80 2.00	5.00 3.60
		Over 39	3	2.8000	0.4000	2.40	3.20
	Memory	18–20	54	3.4889	0.8284	1.00	5.00
		21–30 31–38	46 7	3.2174 3.4571	0.8772 1.0753	1.80 1.60	5.00 4.40
		Over 39	3	3.5333	1.1547	2.20	4.20
Indirect strategies	Metacognitive	18–20	54	3.9370	0.8258	1.40	5.00
		21–30 31–38	46 7	3.7435 3.1143	0.8482 0.7988	1.40 1.40	5.00 3.80
		Over 39	3	3.8667	1.2858	2.40	4.80
	Social	18–20	54	3.9593	0.7803	1.00	5.00
		21–30 31–38	46 7	3.8739 4.0000	0.6441 1.0198	2.40 1.80	5.00 4.80
		Over 39	3	3.5333	0.8083	2.80	4.40
	Affective	18–20	54	3.8815	0.8154	1.00	5.00
		21–30 31–38	46 7	3.6739 3.3429	0.7617 0.8223	2.00 1.80	5.00 4.20
		Over 39	3	3.4667	0.9866	2.80	4.60
Overall strategies		18–20	54	3.7469	0.6653	1.17	5.00
		21–30	46	3.6080	0.6444	2.17	5.00
		31–38	7	3.2905	0.5721	2.10	3.80
		Over 39	3	3.4556	0.7954	2.57	4.10

In terms of the mean score for overall strategies, the 18–20 age group exhibits a higher mean score (3.75) compared to the other groups. The remaining groups are ranked in descending order as follows: 21–30 age group (3.61), over 39 age group (3.46), and 31–38 age group (3.29). When examining the subscales, the 18–20 age group demonstrates the highest mean scores in compensation strategy, metacognitive strategy, and affective strategy, while the 31–38 age group has the highest score in social strategy. Additionally, the over 39 age group exhibits the highest mean score in cognitive strategy.

The results of variance analysis for learners of different ages on each subscale are shown in Table 7.

**Table 7:** Variance analysis of different age groups on each subscale.

	Strategies	*F*-value	*P*-value (Significance)
Direct Strategies	Cognitive	1.482	0.224
	Compensatory	2.940	0.037
	Memory	0.862	0.464
Indirect Strategies	Metacognitive	2.086	0.107
	Social	0.405	0.749
	Affective	1.364	0.258
Overall strategies		1.251	0.295

The analysis of variance shows that the main effect of age on the overall strategies is not significant [*F*(3,106) = 1.251, *p* > 0.05]. This indicates that there is no significant difference in the overall strategies among different age groups. However, the results of the analysis of variance for the subscales show that the main effect of age on the compensatory strategies is significant (*p* < 0.05), which suggests that learners at different age groups have significant differences in their compensatory strategies. In addition, the results of the two-variable correlation analysis show that the correlation between age and overall strategies is not significant (*p* > 0.05), but the correlation between age and compensatory strategies is significant (*p* < 0.05), with a correlation coefficient of –0.240.

The findings of the analysis suggest that there are no significant differences in the overall strategies employed by participants of different ages in their Chinese language learning. Nevertheless, a closer examination of the subscales reveals that there are notable disparities in compensatory strategies among learners belonging to various age groups. The negative correlation coefficient implies that older students score lower on compensatory strategies when compared to their younger counterparts. This discrepancy could be attributed to the fact that younger students may encounter more difficulties comprehending and processing the language, resulting in their reliance on compensatory strategies such as contextual guessing or memorization techniques. Conversely, older students may possess a stronger foundation in Chinese language learning, enabling them to employ more efficient and effective strategies to rectify their weaknesses. In addition to displaying a marked advantage over other age groups in the implementation of compensatory strategies, the 18–20-year-old group also surpasses their peers in terms of metacognitive and affective strategies. Furthermore, they achieve the second highest rankings in the remaining three learning strategies. This indicates that learners within this age range demonstrate a greater level of proactivity and engagement when utilizing learning strategies. We posit that the rationale behind this disparity is linked to learning motivation. In the context of foreign language acquisition, learning motivation plays a crucial role in determining the selection of learning strategies (Oxford and Nyikos, 1989; Oxford, 1990). Individuals with high levels of motivation tend to employ a greater number of strategies compared to those with low motivation, and the strength of motivation and the frequency of employed learning strategies exhibit significant predictive validity for academic achievement. Under the promotion of the “Belt and Road” initiative, all countries are experiencing significant development. The internal driving forces of economic development in various countries have provided young students with learning opportunities and employment prospects. For the subjects, choosing a foreign language to learn may well determine their future.

We conducted interviews with participants belonging to diverse age groups, focusing on three exemplary cases for analysis. Younger learners, like Gu, a 20-year-old female from Kyrgyzstan, frequently cited exams and scholarships as primary motivations, which correlates with our findings that younger learners have higher motivation related to academic and professional goals:

3.   This year is my second year studying Chinese in China. My biggest goal for this year is to pass the HSK Level 4 exam, get a scholarship, and improve my Chinese communication and application skills. This will allow me to communicate effectively in daily life and at work.

Ha, a 21-year-old male from Kyrgyzstan, represents those motivated by career prospects, reflecting a common theme among his age group in our dataset:

4.   Nowadays, lots of companies need workers who can speak Chinese well and understand different cultures to help them grow. So, if we learn Chinese well, we can stand out in these global businesses. Knowing Chinese gives us more job chances and helps us work better with people from other countries and cultures. It also opens up more career paths for us.

Aika, a 36-year-old female learner from Kazakhstan, was chosen to exemplify the older participants who expressed a motivation to learn Chinese for cultural interest, a finding that was less prevalent among younger participants:

5.   My purpose of coming to China to study Mandarin is quite simple: mainly to gain a deeper understanding of Chinese culture. I have a neighbor who studied in China before, and he often tells me some interesting stories about China. Through watching Chinese movies, I developed a strong interest in traditional Chinese culture, cuisine, and Kung Fu. These films have given me more insights into China and ignited my enthusiasm to further explore its culture.

By selecting cases that illustrate these broader trends, we ensure that our exemplary narratives are not merely individual stories but are representative of the larger dataset, offering valuable insights into the varied learning strategies and motivations of male and female language learners across different age groups. This approach allows for targeted teaching strategies that can be more effectively tailored to the needs of different learners within the classroom.

### Correlation between learning durations and learning strategies

It is noteworthy that among the subjects, most of the preparatory students basically have no real experience in learning Chinese before coming to China. Their exposure to Chinese is limited to eating Chinese food, watching Chinese movies, or having visited China before. There are also a few learners who have had Chinese learning experience, but their learning time is relatively short. Most of the master’s and doctoral learners among the subjects have had more than 3 years of Chinese learning experience. In view of the Chinese learning situation, the subjects were divided into four categories according to “less than 1 year,” “1–2 years,” “2–3 years” and “more than 3 years (including 3 years).” Based on this, the differences in the choice of Chinese learning strategies among the subjects due to the length of Chinese learning time are analyzed, as shown in Table 8.

**Table 8:** Mean and standard deviation of learning duration on each subscale.

	Strategies	Age groups	*N*	Mean	Standard deviation	Maximum	Minimum
Direct strategies	Cognitive	Less than 1 year	38	3.6421	0.6757	2.40	5.00
		1–2 years 2–3 years	26 4	3.4538 2.4500	0.6288 1.2261	2.40 2.00	4.60 5.00
		More than 3 years	42	3.3238	0.9443	1.60	5.00
	Compensatory	Less than 1 year	38	3.8895	0.6797	2.40	5.00
		1–2 years 2–3 years	26 4	3.7231 3.5500	0.6477 1.0247	2.40 2.60	5.00 5.00
		More than 3 years	42	3.2714	0.8777	1.00	5.00
	Memory	Less than 1 year	38	3.5421	0.7958	2.20	5.00
		1–2 years 2–3 years	26 4	3.3231 3.1500	0.7901 1.3000	2.00 2.00	5.00 5.00
		More than 3 years	42	3.2762	0.9463	1.00	5.00
Indirect strategies	Metacognitive	Less than 1 year	38	4.2105	0.5807	3.00	5.00
		1–2 years 2–3 years	26 4	3.8615 3.4500	0.7083 1.2689	2.40 2.00	5.00 5.00
		More than 3 years	42	3.4286	0.9562	1.40	5.00
	Social	Less than 1 year	38	4.1105	0.6233	2.40	5.00
		1–2 years 2–3 years	26 4	3.8462 3.7500	0.6127 1.0755	2.60 2.40	5.00 5.00
		More than 3 years	42	3.7952	0.8502	1.00	5.00
	Affective	Less than 1 year	38	4.0053	0.6857	2.60	5.00
		1–2 years 2–3 years	26 4	3.7154 3.4500	0.7487 1.2689	2.40 2.00	5.00 5.00
		More than 3 years	42	3.5667	0.8473	1.00	5.00
Overall strategies		Less than 1 year	38	3.9000	0.5043	3.13	5.00
		1–2 years	26	3.6538	0.5087	2.87	4.80
		2–3 years	4	3.4667	1.1652	2.17	5.00
		more than 3 years	42	3.4437	0.7446	1.17	5.00

The findings of the analysis reveal that there is a significant disparity in the overall strategies utilization for Chinese language learning among participants with varying levels of learning experience. Specifically, the group with less than 1 year of learning experience exhibits the highest mean score (3.90) in terms of overall strategies usage, followed by the 1–2 years of learning experience group (3.65), 2–3 years of learning experience group (3.47), and the group with more than 3 years of learning experience (3.44). The results of the variance analysis concerning learners of diverse learning durations on each subscale are presented in Table 9.

**Table 9:** Variance analysis of different learning durations on each subscale.

	Strategies	*F*-value	*P*-value (Significance)
Direct strategies	Cognitive	1.058	0.370
	Compensatory	4.605	0.005
	Memory	0.762	0.518
Indirect strategies	Metacognitive	6.710	0.000
	Social	1.421	0.241
	Affective	2.302	0.081
Overall strategies			0.017

The results of the analysis of variance show that, in terms of the overall strategies, the main effect of learning duration is significant [*F*(3,106) = 3.558, *p* < 0.05]. This indicates that there are significant differences in the overall strategies between learners with different learning durations. At the same time, the results of the analysis of variance of the subscales show that the main effects of different learning durations on compensation strategies and metacognitive strategies are significant (*p* < 0.05), which indicates that learners with different learning durations have significant differences in compensation strategies and metacognitive strategies. In addition, the results of the two-variable correlation analysis show that the correlation between different learning durations and the overall strategies is relatively significant (*p* < 0.01), with a correlation coefficient of-0.295; it is also significant with compensation strategies and metacognitive strategies, with correlation coefficients of –0.339 and –0.394, respectively, while it is not significant with the other four substrategies.

The analysis results indicate that, on the whole, participants with different learning durations have significant differences in the use of Chinese learning strategies. From the subscale perspective, learners with different learning durations have significant differences in overall strategies, compensatory strategies, and metacognitive strategies. Compared to learners with shorter learning duration, those with longer study time tend to choose fewer compensatory strategies and metacognitive strategies. This suggests that beginner learners may rely more on metacognitive and cognitive strategies to aid their language learning process. As learners gain more experience, they may become more efficient in their language learning strategies and rely less on compensation and social strategies. As can be observed, an increase in study duration leads to a gradual deepening of learners’ comprehension of the characteristics, knowledge system, and learning difficulties inherent in the Chinese language. This enables them to better manage and evaluate their own progress. Consequently, they adjust their learning strategies according to their actual situation, primarily through two aspects: firstly, by reducing metacognitive strategies related to learning process management and coordination; secondly, by reducing compensatory strategies adopted due to a lack of target language knowledge. The findings are not supported by previous research by Oxford and Nyikos (1989). They explored factors affecting choice of language learning strategies among university students and found that students who had studied the language for more than 5 years used functional practice or communicative strategies more frequently than less experienced students. Additionally, students who had spent more than 4 years studying a foreign language used conversational input elicitation strategies more often than less experienced students. To further identify the reasons behind these findings, we conducted interviews with the representative participants. Ai, a 20-year-old female from Kazakhstan who has been pursuing her studies in China for approximately 1 year, provided an insightful account of her initial learning experience:

6.   I just arrived in China to study Chinese, and learning Chinese characters has been really tough for me. Sometimes I use *pinyin* instead of Chinese characters because it’s easier for me to remember and understand. During class, there are times when I cannot catch what the teacher is saying, but I try to guess what they want me to do based on the words I do understand. Even though I feel confused sometimes, I’ve been working hard to learn. I’m really thankful to my teachers who always encourage me to apply what I’ve learned in daily life. This motivates me even more to learn and practice. I listen carefully to my teachers’ explanations and regularly review what I’ve already learned. I believe that if I keep at it, my Chinese will definitely improve.

Am, a 28-year-old male from Kyrgyzstan who has been studying in China for four years and recently passed the HSK6 exam with a commendable level of Chinese proficiency, reflected on how his approach to learning strategies has transformed over time:

7.   When I first started learning Chinese, I had a hard time understanding some words and sentences. I would often guess their meanings, but sometimes I was wrong. However, as my Chinese got better, I could communicate with native speakers more easily and did not have to guess as much. In the beginning, I was strict with myself and made good study habits. I reviewed what I learned regularly and did more exercises to remember it better. I also joined language exchange activities to practice speaking with native speakers. For HSK exams, I always use reasoning and analysis to practice. I read the questions carefully, understand them, and use the information I know to figure out the right answer. At the same time, I try to use the vocabulary and sentence structures I learned from native speakers in my studies and daily life. This helps me not only understand the words and sentences better but also improves my language skills.

Based on the aforementioned analysis, it is evident that an increase in study duration leads to a shift in learners’ choice of learning strategies to accommodate their learning process. Consequently, further attention should be devoted to this matter in order to optimize educational resources and support for students.

## Implications

In this research, we conducted a statistical analysis of the data collected from questionnaires and incorporated the results of face-to-face interviews to propose three teaching strategies for Chinese language learning and instruction among the subjects.

### Personalizing language learning strategies for gender-inclusive education

Based on research findings that reveal no significant gender differences in the utilization of Chinese language learning strategies among the subjects, yet underscore noteworthy disparities in memory and affective strategies, a series of pedagogical recommendations have been posited. These include advocating for personalized learning strategies wherein educators encourage students to select strategies that align with their individual strengths and preferences. For example, whereas female students may exhibit a greater proclivity for employing memory and affective strategies, their male counterparts might exhibit a predilection for cognitive and social strategies. Given the less frequent use of memory strategies among males, augmented memory strategy training could prove advantageous, providing them with additional guidance and practice on efficacious memory techniques. Furthermore, considering the prevalent use of affective strategies among females, educators could devise activities aimed at enabling all students to comprehend and implement affective strategies such as self-encouragement and mental adjustment, thereby augmenting their motivation and confidence in language acquisition.

Educators are also encouraged to endorse a balanced and adaptable application of multiple strategies according to the various learning contexts and objectives. This pedagogical approach acknowledges that rote memorization might be more efficacious when grappling with voluminous information or intricate concepts. Fostering self-directed learning emerges as another pivotal recommendation, inspired by exemplars such as Ho’s case, which underscores the significance of intrinsic motivation and self-drive for effective learning. Consequently, educators should endeavor to cultivate students’ capacities for self-motivation rather than relying excessively on external emotional support. Despite the absence of significant differences in the use of cognitive, compensation, metacognitive, and social strategies between genders, educators must remain vigilant regarding how gender disparities might influence students’ learning attitudes and behaviors, necessitating appropriate modifications in teaching methodologies. Continuous research and observation are also indispensable, particularly since the current study’s findings are premised on a sample from five Central Asian countries. Extended observations of learning strategy utilization among students from diverse cultural and educational backgrounds will deepen our comprehension of gender differences and inform any requisite pedagogical adaptations.

By integrating these suggestions into educational practice, educators can support both male and female students more effectively, assisting them in selecting suitable learning strategies that cater to their unique attributes and requirements, thus enhancing overall learning efficacy and outcomes.

### Personalized instruction and motivational strategies for effective Chinese language education across ages

Although there are no significant differences in overall strategies across various age groups of learners, considerable divergences emerge in compensation strategies. Younger learners often depend more on contextual guessing and memory techniques, whereas their older counterparts might employ different strategies due to a more robust foundation in Chinese. Teachers, therefore, should offer strategy training tailored to students’ ages and language proficiency.

Research has shown that older students tend to score lower on compensation strategies, prompting the recommendation for educators to design specific training for this demographic. This could involve simulation activities and role-playing exercises to enhance their language adaptability. The propensity of the 18–20 age group to utilize metacognitive and affective strategies suggests that these approaches should be encouraged in all age groups to increase initiative and participation in the learning process. Recognizing the critical importance of motivation in language acquisition, it is essential for teachers to develop incentive systems and activities that reinforce the drive to learn, especially among older learners.

With the opportunities presented by the “Belt and Road” initiative, educators should assist students in understanding how learning Chinese can impact their future career development, encouraging them to integrate language learning with professional aspirations. In light of some learners’ interest in Chinese culture, educators could benefit from organizing cross-cultural exchanges such as film viewings, culinary experiences, and martial arts practice, which can deepen cultural insights and invigorate learning passion. Success stories like those of Gu, Ha, and Aika can serve as case studies to demonstrate the practical application of language skills in real-life and work environments, thereby inspiring a more active approach to learning.

In conclusion, adjusting teaching methods to align with the traits of different age groups of learners is crucial. Emphasis should be placed on teaching compensation strategies, fostering motivation, and incorporating cross-cultural activities to make learning both engaging and applicable.

### Strategic approaches to enhance Chinese language learning for diverse learners

To enhance the effectiveness of Chinese language education across various age groups and learning stages, it is crucial for educators to implement stage-based strategy training that aligns with the learners’ duration of study. Beginners should be primarily equipped with metacognitive and cognitive strategies to manage and coordinate their learning process effectively. As learners advance, they tend to rely less on compensation and social strategies, opting instead for more efficient learning approaches. Personalized learning plans become increasingly important as students gain a deeper comprehension of the language’s characteristics and learning challenges, allowing them to adjust their strategies to fit their actual situation.

Moreover, practical application and constant practice are paramount. Teachers should encourage students to apply learned material in real-life contexts and participate in language exchange activities to enhance their communication skills. For those preparing for exams like HSK, targeted strategy training in reasoning and analysis can prove beneficial.

Continuous motivation and support from teachers play a significant role in fostering a positive learning environment. Institutions must provide regular feedback and recognize students’ efforts to keep them motivated. Finally, educational institutions should consider the discrepancies between current findings and previous research, such as that of Oxford and Nyikos (1989), and engage in further studies to optimize resources and support for diverse learners. By doing so, educators can ensure that teaching strategies evolve to meet the individual needs of each student, thereby facilitating their Chinese language learning journey effectively.

## Conclusion

This mixed methods study has illuminated the nuanced ways in which Chinese language learners from Central Asian countries adopt different learning strategies based on their gender, age, and duration of study. The overarching finding is that female learners are more inclined towards memory and affective strategies, whereas their male counterparts show a preference for cognitive and problem-solving approaches. This dichotomy underscores the necessity for gender-sensitive teaching methodologies within Chinese language education for this population. Furthermore, our analysis has revealed that age plays a significant role in shaping learning strategy preferences, with older students demonstrating superior adoption of effective language learning strategies compared to their younger peers. Notably, the 18–20 age group excelled in compensatory, metacognitive, and affective strategies, pointing towards their heightened proactivity and engagement in the learning process. This suggests that instructional approaches should be flexibly tailored to cater to the varied needs of different age groups. Learning duration was also found to be a critical determinant, with beginner learners showing a greater reliance on compensatory, metacognitive, and cognitive strategies, indicating that experience influences the efficiency and approach to language learning. Educators can leverage these insights to offer targeted pedagogical strategies that align with students’ learning stages.

Despite providing valuable insights, this study is not without limitations. A key limitation is the sample size, which may not fully represent the diversity of learners from Central Asian countries studying Chinese. Future research efforts should aim to include a broader and more representative sample to validate and extend our findings. Additionally, the reliance on self-reported data and semi-structured interviews introduces potential biases. To address this, future studies could incorporate multiple data sources, including observations and in-depth interviews with educators and peers. Moreover, while this study focused on demographic factors such as gender, age, and learning duration, other influential factors like motivation, cultural background, and prior language learning experiences were not thoroughly explored. Comprehensive future research should aim to unravel the complex interplay between these variables and learning strategies in the context of Chinese language acquisition.

The results have significant implications for educational practice, guiding the design of more inclusive and personalized curricula. Furthermore, the findings contribute to the larger goal of fostering cross-cultural communication and collaboration, especially pertinent to the Belt and Road Initiative, by enhancing language skills among learners from Central Asia.

## Data availability statement

The original contributions presented in the study are included in the article/supplementary material, further inquiries can be directed to the corresponding author.

## Ethics statement

The study involving human participants was reviewed and approved by the Ethics Committee of Xuchang University. The studies were conducted in accordance with the local legislation and institutional requirements. The participants provided their written informed consent to participate in this study.

## Author contributions

RC: Conceptualization, Writing – original draft, Writing – review & editing. LZ: Data curation, Investigation, Methodology, Writing – review & editing.

## Footnotes

^1^“Learning duration” refers to the amount of time a learner has been engaged in the process of learning a particular subject, skill, or language. In the context of this research on Chinese learning strategies employed by learners from Central Asian countries, “learning duration” specifically pertains to the period an individual has been studying Chinese as a foreign language. It involves the time spent in formal education settings, self-study, and any other forms of language learning experiences that contribute to the acquisition of Chinese language skills.

## Appendix I

The contents of the questionnaire.

1. I always take notes diligently in class.
2. I constantly imitate the pronunciation and intonation of Chinese people.
3. I often watch Chinese TV programs or movies, noting down useful sentences.
4. I often practice writing notes and letters in Chinese to improve your Chinese writing skills.
5. I frequently summarize the Chinese information read and heard, striving to remember it.
6. When encountering new vocabulary, I always guess its meaning first.
7. When reading and listening to texts, I can understand the general idea even with some new words.
8. I always break down Chinese words into known parts to guess their meanings.
9. Encountering new words in spoken expression, I will ask the people around.
10. When unable to recall a word in spoken expression, I use body language or similar words to express.
11. I am able to listen to the class very attentively and focusedly.
12. I arrange my time reasonably, ensuring enough time to learn Chinese.
13. I have a clear goal for improving my Chinese proficiency.
14. I always strive to find opportunities to practice and use Chinese.
15. After class, I am able to review and practice Chinese well.
16. I remember the pronunciation of new Chinese words by combining it with its writing or graphics.
17. I demonstrate newly learned vocabulary through gestures and actions.
18. I practice new vocabulary through sentence making.
19. I carry cards with new words and sentences learned for review.
20. I do not memorize vocabulary in isolation; always within sentences.
21. I believe that communicating with Chinese people is important for learning Chinese.
22. I often exchange and practice Chinese with others who are learning the language.
23. I ask native Chinese speakers to correct my grammar and vocabulary mistakes.
24. When unable to understand Chinese spoken by others, I ask them to speak slower or repeat.
25. I often communicate in Chinese with classmates, helping each other improve.
26. I always encourage myself to use Chinese as much as possible.
27. I try to relax before speaking Chinese when nervous.
28. Small mistakes do not prevent me from continuing to express in Chinese.
29. I reward myself when making progress in learning Chinese.
30. I share experiences of learning Chinese with other learners.

## Appendix II

The specific questions for the interview.

1. Could you share any tips on how to recognize when simple memorization might be the most practical approach?
2. How do you create mental linkages when trying to remember new information?
3. Could you share some examples of how you apply images and sounds to aid memorization?
4. Could you give an example of how practical application has deepened your understanding of a subject?
5. Can you provide an instance where practicing has helped you understand a language concept better?
6. Could you discuss a situation where analyzing and reasoning facilitated your comprehension of language material?
7. In what situations have you found intelligent guessing to be beneficial?
8. Could you describe a scenario where you overcame limitations in speaking or writing while learning a new language?
9. How do you go about creating a study plan, and what elements does it typically include?
10. Can you discuss a time when self-testing helped you assess your understanding and progress?
11. Could you provide specific examples of how you engage in thinking, exploration, and practice while learning?
12. How do you manage expectations to reduce anxiety related to language learning?
13. Can you give an example of how self-praise or positive self-talk has encouraged your self-confidence in language learning?
14. How do you maintain a positive attitude towards learning by focusing on the benefits and enjoyment of language learning?
15. Why do you believe that communication and interaction with others are crucial for understanding and applying knowledge?
16. Could you provide an example of how imitating native speakers has improved your fluency?
17. Could you give an example of how practical application has deepened your understanding of a subject?
18. How would you describe your unique learning style, and how does it influence your approach to learning?
19. Can you discuss how finding the right learning method has improved your learning effectiveness?
20. When faced with a large amount of information, how do you decide which learning strategies to use for optimal efficiency?

## References

[ref2] Alhaysony M. (2017). Language learning strategies use by Saudi EFL students: the effect of duration of English language study and gender. Theory Pract. Lang. Stud. 7:18. doi: 10.17507/tpls.0701.03

[ref3] Al-Khazale B. A. (2019). Exploring language learning strategies of Saudi EFL learners at Shaqra University, Saudi Arabia. Adv. Lang. Liter. Stud. 10, 63–71. doi: 10.7575/AIAC.ALLS.V.10N.4P.63

[ref4] Aslan O. (2009). The role of gender and language learning strategies in learning English Master's thesis, Middle East Technical University.

[ref38] Bacon S. M. (1992). The relationship between gender, comprehension, processing strategies, and cognitive and affective response in foreign language listening. Mod. Lang. J. 76, 160–178. doi: 10.1111/j.1540-4781.1992.tb01096.x

[ref5] Bialystok E. (1978). A theoretical model of second language learning. Lang. Learn. 28, 69–83. doi: 10.1111/j.1467-1770.1978.tb00305.x

[ref9002] Bialystok E. (1997). The structure of age: in search of barriers to second language acquisition. Second Lang. Res. 13, 116–137. doi: 10.1191/026765897677670241

[ref9003] Brown T. A. (2006). Confirmatory factor analysis for applied research. New York: Guilford Press.

[ref6] Chai C. S. Wong L. H. King R. B. (2016). Surveying and modeling students’ motivation and learning strategies for mobile assisted seamless Chinese language learning. Educ. Technol. Soc. 19, 170–180,

[ref7] Chang Y. P. (2003). Factors affecting language learning strategy choice: a study of EFL senior high school students in Taiwan. Doctoral dissertation, Texas: A & M University - Kingsville.

[ref8] Chen M. L. (2014). Age differences in the use of language learning strategies. Engl. Lang. Teach. 7, 144–151. doi: 10.5539/elt.v7n2p144

[ref9] Chou M. H. (2018). Speaking anxiety and strategy use for learning English as a foreign language in full and partial English-medium instruction contexts. TESOL Q. 52, 611–633. doi: 10.1002/tesq.455

[ref10] Corbin A. (2017). Assessing differences in learning styles: age, gender and academic performance at the tertiary level in the Caribbean. Caribb. Teach. Scholar 7, 67–91,

[ref11] Ding X. (2016). Exploring the experiences of international students in China. J. Stud. Int. Educ. 20, 319–338. doi: 10.1177/1028315316647164

[ref12] Dinsa M. T. Seyoum G. Dinsa D. T. (2022). The influence of gender and study duration on EFL learners’ speaking strategies use. Int. J. Lang. Educ.. 6, 10–24. doi: 10.26858/ijole.v6i1.19272

[ref13] Ehrman M. Oxford R. (1989). Effects of sex differences, career choice, and psychological type on adult language learning strategies. Mod. Lang. J. 73, 1–13. doi: 10.2307/327261

[ref14] Gong Y. Gao X. Lyu B. (2020). Teaching Chinese as a second or foreign language to non-Chinese learners in mainland China (2014-2018). Lang. Teach. 53, 44–62. doi: 10.1017/S0261444819000387

[ref15] Griffiths C. (2003). Patterns of language learning strategy use. System 31, 367–383. doi: 10.1016/S0346-251X(03)00048-4

[ref9009] Griffiths C. (2008). Lessons from good language learners. Cambridge: Cambridge University Press.

[ref16] Ibrahim M. Soepriadi D. N. Limbong S. Sujarwo S. Sasabone L. (2023). Non EFL students’ perception in English language learning strategies (LLS) in the digital era. AL-ISHLAH 15, 587–596. doi: 10.35445/alishlah.v15i1.2687

[ref17] Khalil A. (2005). Assessment of language learning strategies used by Palestinian EFL learners. Foreign Lang. Ann. 38, 108–117. doi: 10.1111/j.1944-9720.2005.tb02458.x

[ref18] Khamkhien A. (2010). Factors affecting language learning strategy reported usage by Thai and Vietnamese EFL learners. Electron. J. Foreign Lang. Teach. 116, 2332–2335. doi: 10.1002/ange.200353562

[ref20] Li C. Chen L. Ma C. Zhang S. Huang H. (2021). Strategy use among Chinese as second language learners in mainland China from the mediation theory perspective. Front. Psychol. 12:752084. doi: 10.3389/fpsyg.2021.752084, PMID: 34721232 PMC8548457

[ref9001] Macaro E. (2006). Strategies for language learning and for language use: Revising the theoretical framework. Mod. Lang. J. 90, 320–337. doi: 10.1111/j.1540-4781.2006.00425.x

[ref21] Melvina Lengkanawati N. S. Wirza Y. (2020). EFL learners’ language learning strategies: field specialization and gender. Int. J. Educ. 13, 63–69. doi: 10.17509/ije.v13i2.20972

[ref22] Milla R. Gutierrez-Mangado M. J. (2019). Language learning strategy reported choice by bilingual children in CLIL: the effect of age, proficiency and gender in L3 learners of English. System 87:102165. doi: 10.1016/j.system.2019.102165

[ref23] Min T. A. Min O. Y. Qi C. P. Yoon C. L. Mei L. S. Rahmat N. H. (2022). Exploring strategies in language learning: the case for mandarin as a foreign language. Int. J. Acad. Res. Bus. Soc. Sci.. 12, 1592–1610. doi: 10.6007/IJARBSS/v12-i11/14847

[ref25] Montero-SaizAja A. (2021). Gender-based differences in EFL learners’ language learning strategies and productive vocabulary. Theory Pract. Second Lang. Acquisit. 7, 83–107. doi: 10.31261/TAPSLA.8594

[ref26] My N. T. T. Dan T. C. (2023). EFL students’ perceptions and practices on learning strategies towards ESP for political education: a case at school of political science, can Tho University, Vietnam. Eur. J. Alternat. Educ. Stud. 8, 47–91. doi: 10.46827/ejae.v8i2.4823

[ref27] Nguyen N. Godwyll F. (2010). Factors influencing language-learning strategy use of English learners in an ESL context. Mid-Western Educ. Research. 23, 7–13.

[ref9005] Nyikos M. (1990). Gender-related differences in adult language learning: Socialization and memory factors. Mod. Lang. J. 74, 273–287. doi: 10.1111/j.1540-4781.1990.tb01063.x

[ref9006] O’Malley J. M. Chamot A. U. (1990). Learning strategies in second language acquisition. Cambridge: Cambridge University Press.

[ref9007] Ortega L. (2009). Understanding second language acquisition. Oxford University Press.

[ref28] Oxford R. L. (1989). Use of language learning strategies: a synthesis of studies with implications for strategy training. System 17, 235–247. doi: 10.1016/0346-251X(89)90036-5

[ref29] Oxford R. L. (1990). Language learning strategies: what every teacher should know. TESOL Q. 27:121. doi: 10.2307/3586958

[ref30] Oxford R. L. (2013). Teaching and researching language learning strategies. New York, NY: Routledge.

[ref31] Oxford R. L. Nyikos M. (1989). Variables affecting choice of language learning strategies: a synthesis of studies with implications for strategy training. System. 17, 235–247.

[ref9008] Pressley M. McCormick C. B. (1995). Advanced educational psychology: for educators, researchers, and policymakers. New York: Harper Collins College Publishers.

[ref32] Psaltou-Joycey A. Mattheoudakis M. Alexiou T. (2014). “Language learning strategies in CLIL and non-CLIL classes: which strategies do young learners claim they use?” in Cross-curricular approaches to language education. eds. Psaltou-Joycey A. Agathopoulou E. Mattheoudakis M. (UK: Cambridge Scholars Publishing), 305–322.

[ref33] Radwan A. A. (2011). Effects of L2 proficiency and gender on choice of language learning strategies by university students majoring in English. Asian EFL J. 13, 114–162,

[ref34] Rafik-Galea S. Wong B. E. Chan W. Chin K. Suthiwan T. (2011). Vocabulary learning strategies among adult foreign language learners. In Foreign Language Teaching in Asia and Beyond: current Perspectives and Future Directions. Chan W. M. Chin K. N. Suthiwan T. Eds. (Berlin: De Gruyter Mouton). 145–188.

[ref35] Rubin J. (1975). What the “good language learner” can teach us. TESOL Q. 9:41. doi: 10.2307/3586011

[ref36] Shi J. Mo X. Sun Z. (2012). Content validity index in scale development. Zhong nan da xue xue bao. J. Central South Univ. 37, 152–155. doi: 10.3969/j.issn.1672-7347.2012.02.007, PMID: 22561427

[ref37] Sumarni S. Rachmawaty N. (2019). Gender differences in language learning strategies. Ethical Lingua. 6, 13–22. doi: 10.5281/zenodo.7972543

[ref39] Szyszka M. (2017). Pronunciation learning strategies and language anxiety, vol. 10. Switzerland: Springer, 978–973.

[ref40] Vidal R. T. (2002). Is there a correlation between reported language learning strategy use, actual strategy use and achievement? Revista Linguagem Ensino 5, 43–73. doi: 10.15210/rle.v5i1.15550

[ref41] Wang Y. (2017). Language policy in Chinese higher education: a focus on international students in China. Eur. J. Lang. Policy 9, 45–66. doi: 10.3828/ejlp.2017.4

[ref9011] Yaman S. Ozcan M. (2015). Oral communication strategies used by Turkish students learning English as a foreign language. Issues in teaching, learning and testing speaking in a second language, 143–158. doi: 10.1007/978-3-642-38339-7_9

[ref44] Zhang L. Sun S. Yao S. (2022). Error types of and strategies on learning Chinese connectives: a study on Chinese as a second language learners’ writing. Front. Psychol. 12:790710. doi: 10.3389/fpsyg.2021.790710, PMID: 35140659 PMC8819010

[ref45] Zhang L. Tsung L. (2021). Learning Chinese as a second language in China: positive emotions and enjoyment. System 96:102410. doi: 10.31838/jcr.07.06.141

[ref46] Zhang L. Wan Y. (2019). On the Chinese learning strategies employed by foreign students from five Central Asian countries. Chin. Lang. Learn. 2, 94–104,

